# Combined Conventional Blood Biomarkers as Discriminators of Excessive Alcohol Consumption in Men: A Large-Scale Cross-Sectional Study

**DOI:** 10.3390/healthcare14030394

**Published:** 2026-02-04

**Authors:** Ichiro Wakabayashi

**Affiliations:** Department of Preventive Medicine, School of Medicine, Hyogo Medical University, Nishinomiya, Hyogo 663-8501, Japan; wakabaya@hyo-med.ac.jp; Tel.: +81-798-45-6561

**Keywords:** alcohol, biomarker, gamma glutamyl transferase, HDL cholesterol, mean corpuscular volume

## Abstract

**Background/Objectives**: Blood biomarkers for estimating alcohol consumption are useful for preventing alcohol-related harms. Although there are conventional blood biomarkers of heavy alcohol drinkers, it remains to be clarified whether their combination is useful for estimation of excessive alcohol consumption from the viewpoint of preventing hypertension. **Methods**: Participants included 8172 men aged from 31 to 70 years who had undergone health checkups. Overall, participants were classified into three groups of nondrinkers, occasional drinkers, and regular drinkers by frequency; regular drinkers were further classified into four groups of light (<22 g/day), moderate (≥22 and <44 g/day), heavy (≥44 and <66 g/day), and very heavy drinkers (≥66 g/day) according to the amount of average daily alcohol consumption. The relationships of blood biomarkers (mean corpuscular volume [MCV], gamma glutamyl transferase [GGT], and HDL cholesterol [HDL-C]) and their products with alcohol consumption were investigated by using correlation analysis and receiver-operating characteristics (ROC) analysis. **Results**: Seven variables of blood biomarkers and their products were significantly correlated with frequency and amount of alcohol consumption, and the degrees of the correlations were stronger in the following order: HDL-C alone < product of MCV and HDL-C < MCV alone < GGT alone < product of MCV and GGT < product of GGT and HDL-C < product of MCV, HDL-C and GGT. In the ROC analysis, the area under the ROC curve for the relationship between the product of MCV, HDL-C, and GGT (named the alcohol consumption index [ACI]) and excessive alcohol intake (22 g/day or more) was 0.819 (95% confidence interval: 0.809–0.830), and the cutoff of this index was 194,863 with a sensitivity and specificity of 0.745 and 0.751, respectively. The positive predictive value was 69.2%. **Conclusions**: Among the three conventional blood biomarkers and their combinations, ACI demonstrated the strongest associations with alcohol consumption and excessive alcohol intake in men. Although the combined biomarkers are unlikely to be useful as a diagnostic tool, there is a possibility of future application by integrating ACI with recent biomarkers including carbohydrate-deficient transferrin for estimation of alcohol consumption.

## 1. Introduction

Excessive alcohol consumption reduces life expectancy by causing various diseases including cancer, liver cirrhosis, and hemorrhagic stroke as well as exogenous causes of death such as traffic accidents and suicide [1]. Conversely, low alcohol consumption is associated with lower mortality, which is believed to be mainly due to the decreased incidence of ischemic heart disease in older populations [2,3]. Thus, knowing the amount of individual habitual alcohol consumption is helpful when instructing patients to improve their alcohol drinking behavior. Conventional blood biomarkers including gamma glutamyl transferase (GGT), mean corpuscular volume (MCV), and HDL cholesterol (HDL-C), have been investigated to estimate chronic alcohol consumption. Their validity as biomarkers is determined by their correlations with alcohol intake and by reductions in elevated levels following abstinence in heavy drinkers [4]. In fact, GGT, MCV, and HDL-C levels have been reported to decrease after one month of alcohol abstinence and increase after one month of moderate alcohol consumption in healthy men [5]. These markers of heavy alcohol consumption were reportedly predictive of medical sequelae and healthcare utilization in men attending the ambulatory care services [6]. Among 25 biochemical and hematological measurements, GGT was reported to show the strongest association with alcohol consumption [7]. Conversely, a previous study revealed that alcohol consumption was associated with MCV and HDL-C, but not with GGT in healthy men [8].

Regarding the validity of the markers, abnormal values of GGT, MCV, and HDL-C had low sensitivity but high specificity for identifying drinking problems in ambulatory patients [9]. In patients with alcoholism, GGT levels identified regular heavy drinkers with a sensitivity of 40–60%, whereas MCV had a high specificity of 95% [10]. For the objective diagnosis of alcohol abuse, the reported sensitivity and specificity were 24% and 96% for MCV, respectively, and 42% and 76% for GGT, respectively [11]. In participants recruited from the community and alcohol dependence treatment services, GGT showed a sensitivity and specificity of 67% and 74%, respectively, for heavy drinking (>80 g/day) in men [12]. Despite the accumulated knowledge, blood biomarkers for estimating alcohol consumption, which are useful for preventing alcohol-related harms, have not yet been confirmed. Biomarker characteristics such as sensitivity and specificity have mainly been investigated in patients with alcohol use disorder (AUD). There is limited knowledge regarding the association between biomarkers and alcohol intake in healthy individuals.

Hypertension is a major risk factor for cerebrovascular and cardiovascular diseases [13], and habitual alcohol consumption is an important modifiable risk factor for hypertension [14,15]. According to the American College of Cardiology/American Heart Association (ACC/AHA) guideline, alcohol intake should be restricted to <20 g in men and <10 g in women to prevent hypertension [13]. However, to the best of my knowledge, there have been no reports on possible biomarkers to distinguish alcohol drinkers who consume higher amounts of alcohol than the above-mentioned border, from the viewpoint of hypertension prevention. Moreover, there is limited information on the relationship between combined biomarkers and alcohol consumption in healthy cohorts because most previous works focused on patients with AUD rather than healthy individuals.

Therefore, this study aimed to investigate the relationships of conventional blood biomarkers—including GGT, MCV, and HDL-C—and their combination with alcohol consumption; it also aimed to determine whether they are useful for the estimation of alcohol consumption and detection of excessive alcohol consumption in drinkers with a high risk of hypertension in a general population.

## 2. Materials and Methods

### 2.1. Participants

The participants of this study included 8172 Japanese men (median age in years with interquartile range: 50 [44–56] years) who had undergone periodic health checkup examinations at workplaces in Yamagata Prefecture, Japan. This study was approved by the Ethics Committee of Hyogo College of Medicine (No. 3003 in 2020), and informed consent was not required for this study because it used de-identified secondary data from the All Japan Labour Welfare Foundation. History of alcohol consumption, cigarette smoking, regular exercise, illness, and therapy for illness was surveyed using questionnaires. Those with a history of therapy for anemia, liver diseases, and/or dyslipidemia were excluded from the analysis because these conditions considerably influence the levels of MCV, GGT, and HDL-C.

Average alcohol consumption per week was recorded in the questionnaire. Frequency of habitual alcohol consumption was asked in the questionnaire as “How frequently do you drink alcohol?”. The frequency of weekly alcohol consumption was categorized as “every day” (regular drinkers), “sometimes” (occasional drinkers) and “never” (nondrinkers). The next question was “What amount of alcohol per day do you consume?”. In the questionnaire, drinkers were classified by using “go”, a traditional Japanese unit of sake (rice wine). One “go” corresponds to approximately 22 g of ethanol. The amounts of other alcoholic beverages, including beer, wine, whisky, and shochu (a traditional Japanese distilled spirit), were converted and expressed as units of “go”. One go corresponded to approximately 180 mL of sake, 500 mL of beer, 240 mL of wine, 60 mL of whisky, and 80 mL of shochu. The response categories for the above question on amount of alcohol intake were “null”, “less than one go (about 22 g of alcohol)”, “one go or more and less than two go”, “two go or more and less than three go”, and “three go or more”, which were corresponded to nondrinker, light drinker, moderate drinker, heavy drinker and very heavy drinker, respectively. Individuals who drank excessive amounts of alcohol were defined as those consuming one go or more (≥22 g) of alcohol per day, and were included in moderate, heavy, and very heavy drinker groups.

Regarding smokers, in the self-written questionnaire paper, participants were first asked “Are you a habitual cigarette smoker?” Cigarette smokers were defined as participants who had smoked for 6 months or longer and had smoked for the past month or longer. Then, the participants who were smokers were further asked “What is your average cigarette consumption per day?”. The response categories for this question were “20 or fewer cigarettes per day”, “21 or more and 40 or fewer cigarettes per day” and “41 or more cigarettes per day”. Because the percentage of participants who consumed very heavy cigarette consumption (41 or more cigarettes per day) was very low (0.44% [n = 36]), they were included in the heavy smoking group. Consequently, the participants were divided into three groups of nonsmokers, light smokers (≤20 cigarettes per day), and heavy smokers (≥21 cigarettes per day). Participants with regular exercise habits were defined as those exercising almost every day for 30 min or longer per day.

### 2.2. Measurements

Body weight was measured with light clothes during the health checkup. Body mass index (BMI) was calculated as weight in kilograms divided by the square of height in meters. Blood pressure was measured on the day of the health checkup after each subject had rested quietly in a sitting position. Hypertension was defined as systolic blood pressure of ≥140 mmHg and/or diastolic blood pressure of ≥90 mmHg. In addition, individuals who were receiving drug therapy for hypertension were included in the hypertensive group regardless of blood pressure levels. Fasting blood samples were collected from each participant in the morning. Erythrocyte count and hematocrit were measured using the direct current sheath flow detection method and erythrocyte high pulse wave detection method, respectively. Serum concentrations of HDL-C and GGT were measured using enzymatic methods with the commercial kits, cholestest N-HDL and Pureauto S γ-GT (Sekisui Medical Co., Ltd., Tokyo, Japan), respectively.

### 2.3. Statistical Analysis

Continuous variables were summarized as means with standard deviations (or errors) or as medians with interquartile ranges, as appropriate. Categorical variables were summarized as frequencies and proportions. In the correlation analysis, Spearman’s rank correlation coefficients of each biomarker variable with the frequency and amount of alcohol consumption were calculated. The means of each variable were compared in different drinker category groups using analysis of covariance (ANCOVA) with adjustments for age, body weight (or BMI), and habits of smoking and regular exercise. As the accurate average daily alcohol consumption of occasional drinkers was difficult to know, occasional drinkers (n = 2632) were excluded from being subjects for the analysis of the relationships of each variable with the amount of alcohol consumption and excessive alcohol consumption.

Receiver operating characteristic (ROC) analysis was conducted to investigate the relationship between each variable and excessive alcohol consumption. The area under the ROC curve (AUC) and 95% confidence interval were estimated empirically. The optimal cutoff points for the product of MCV, GGT, and HDL-C (named alcohol consumption index [ACI] in this study) were obtained using excessive alcohol consumption (22 g of alcohol per day or more) as the outcome. The optimal cutoff point was selected by maximizing Youden’s index, which is the difference between the true positive rate (sensitivity) and false positive rate (1-specificity) in the ROC curve.

The proportions of individuals showing high ACI levels (≥194,863) were compared using logistic regression analysis, in which the crude and adjusted odds ratios were estimated with their corresponding 95% confidence intervals. In the multivariable analyses, age, body weight, and the current histories of smoking and regular exercise were adjusted for.

All probability (*p*) values were two-sided. Statistical significance was defined as a *p*-value < 0.05. A computer software program (IBM SPSS Statistics for Windows, Version 25.0. IBM Corp, Armonk, NY, USA) was used for statistical analyses.

## 3. Results

The participants’ characteristics are listed in Table 1. When the participants were classified by frequency of alcohol consumption, 70.3% were drinkers, and the proportions of occasional and regular drinkers were 32.2% and 38.1%, respectively. In the group including nondrinkers and regular drinkers (excluding occasional drinkers), the proportions of light drinkers, moderate drinkers, heavy drinkers, and very heavy drinkers were 13.3%, 29.2%, 11.8%, and 2.0%, respectively.

**Figure 1:**
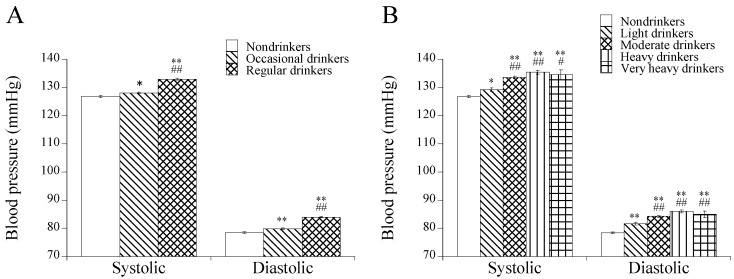
Comparison of blood pressure levels in drinker groups categorized by frequency (**A**) and amount of alcohol consumption (**B**). Means with standard errors are shown. Adjustment was performed for age, BMI, habits of smoking and regular exercise, and a history of medication therapy for hypertension. Symbols denote significant differences from nondrinkers (*, *p* < 0.05; **, *p* < 0.01) and occasional or light drinkers (#, *p* < 0.05; ##, *p* < 0.01).

Three categories of frequency of alcohol intake (non, occasional, and regular drinkers) and five categories of the amount of alcohol consumption (non, light, moderate, heavy, and very heavy drinkers) were used for comparison. Systolic and diastolic blood pressure levels tended to be higher with increased frequency and amount of alcohol consumption; they were significantly higher in regular drinkers compared to nondrinkers and occasional drinkers and were significantly higher in moderate, heavy, and very heavy drinkers (≥22 g/day) compared to nondrinkers and light drinkers (<22 g/day) (Figure 1). Similar results were obtained regarding the prevalence of hypertension: 32.2% (nondrinkers) vs. 36.4% (occasional drinkers) vs. 49.7% (regular drinkers) (*p* < 0.01); 32.2% (nondrinkers) vs. 36.7% (light drinkers) vs. 51.3% (moderate drinkers) vs. 60.6% (heavy drinkers) vs. 46.4% (very heavy drinkers) (*p* < 0.01). Thus, the endpoint of ≥22 g/day is clinically meaningful from the view point of the prevention of hypertension.

Table 2 reveals comparisons of each biomarker in the groups classified by frequency or amount of alcohol consumption. Both MCV, GGT, and HDL-C tended to be higher with increased frequency and amount of alcohol consumption. The results of correlation analysis are shown in Table 3. All of the variables of the tested markers demonstrated significant correlations with both frequency and amount of alcohol consumption. The correlations with the amount of alcohol consumption were stronger than those with frequency of alcohol consumption. The degrees of the correlations of the seven variables with frequency and amount of alcohol consumption were stronger in the following order: HDL-C < product of MCV and HDL-C < MCV < GGT < product of MCV and GGT < product of GGT and HDL-C < product of MCV, GGT, and HDL-C.

**Figure 2:**
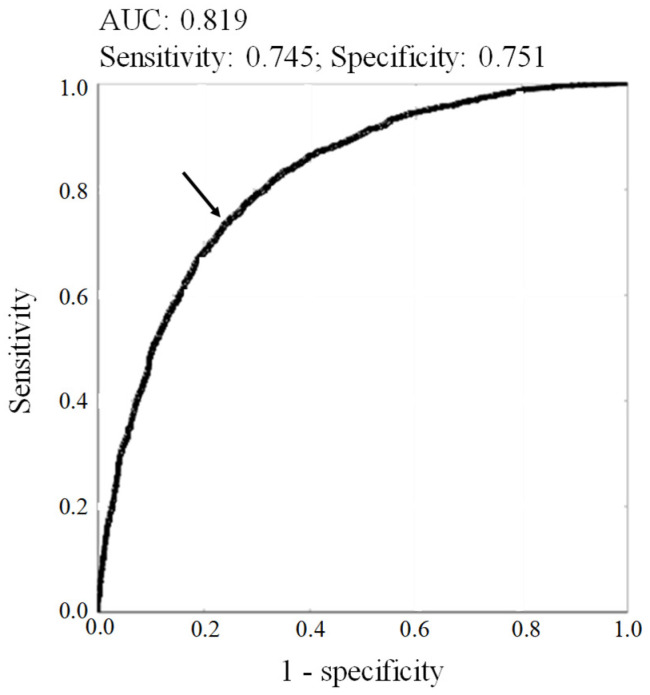
Receiver operating characteristics (ROC) curve for the relationship between ACI and excessive alcohol consumption. The arrow in the figure indicates the cutoff point. Area under the ROC curve (AUC) and sensitivity and specificity of the cutoff point are shown in the figure.

Table 3 also shows the results of the ROC analysis for the relationship between each variable and excessive alcohol consumption defined as alcohol consumption of 22 g/day or more from the viewpoint of prevention of hypertension. The AUCs showed significant relationships between all variables and excessive alcohol consumption. The AUCs for the variables were in the following order: HDL-C < product of MCV and HDL-C < MCV < GGT < product of MCV and GGT < product of GGT and HDL-C < product of MCV, HDL-C and GGT. Thus, in the correlation analysis and ROC analysis, the product of MCV, GGT, and HDL-C showed the strongest relations with frequency and amount of alcohol consumption and excessive alcohol intake. This value was named the ACI (alcohol consumption index) in this study. The cutoff of ACI for excessive alcohol intake was estimated to be 194,863 with the sensitivity and specificity of 0.745 and 0.751, respectively (Figure 2).

Using the Youden index cutoff of ACI (194,863), 3566 participants (43.6% of total participants [n = 8172]) were regarded as individuals with excessive alcohol consumption. In the groups of nondrinkers and regular drinkers (n = 5540) excluding occasional drinkers, 1772 participants showed ACI levels of 194,863 or higher and corresponded to 69.2% of 2560 participants who were detected as those with excessive alcohol consumption (≥22 g/day) evaluated by using the questionnaires. Thus, the positive predictive value was 69.2% when the Youden index cutoff was used for the relationship between ACI and excessive alcohol consumption.

**Figure 3:**
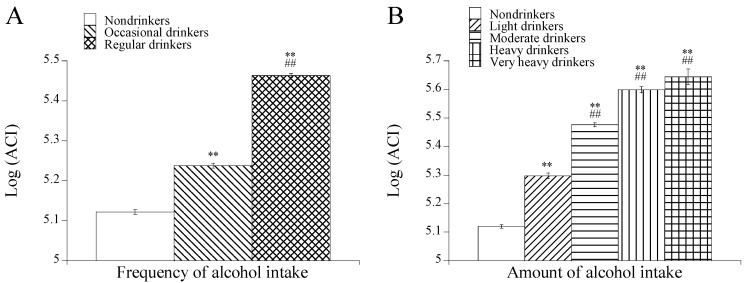
Comparison of log-transformed ACI levels in drinker groups categorized by frequency (**A**) and amount of alcohol consumption (**B**). Means with standard errors are shown. Adjustment was performed for age BMI, and habits of smoking and regular exercise. Symbols denote significant differences from nondrinkers (**, *p* < 0.01) and occasional or light drinkers (##, *p* < 0.01).

When this value (194,863) of ACI was used as a cutoff, the crude odds ratio for excessive alcohol intake in participants with high ACI compared with those without high ACI was estimated to be 8.77 with a 95% confidence interval between 7.76 and 9.92. This association was not altered in the multivariable analysis with adjustment for age, body weight and habits of smoking and regular exercise (odds ratio: 8.30 [7.32–9.41]). In the multivariable analysis with adjustment for age, BMI, and histories of smoking and regular exercise, the mean log-transformed ACI tended to be higher with increases in frequency and amount of alcohol intake (Figure 3). Therefore, the association between ACI and alcohol intake was independent of age, BMI, smoking, and regular exercise.

The relationship between ACI and heavier alcohol consumption was also investigated using ROC analysis. AUC for participants with alcohol consumption of ≥44 g/day and ≥66 g/day were 0.788 and 0.770, respectively, and corresponding cutoff values of ACI were 219,467 (sensitivity: 0.801; specificity: 0.654) and 310,679 (sensitivity: 0.682; specificity: 0.736). The positive predictive values for alcohol consumption of ≥44 g/day and ≥66 g/day were 27.0% and 5.0%, respectively.

## 4. Discussion

### 4.1. Clinical Relevance

When conventional blood biomarkers were combined and used as a single marker, they showed stronger associations with alcohol consumption than individual biomarkers. In particular, the association of the product of GGT and HDL-C with alcohol consumption was stronger than that of the product of MCV and GGT or the product of MCV and HDL-C. Since the product of all three biomarkers (ACI) showed the strongest associations with both alcohol consumption in correlation analysis and excessive alcohol intake in ROC analysis, ACI was used for further analysis to determine the cutoff value for excessive alcohol intake (≥22 g/day). Using this ACI cutoff, the odds ratio for excessive alcohol intake of participants with vs. those without a high ACI was 8.30. This is the first study to demonstrate the relationships between combined blood biomarkers and excessive alcohol consumption from the viewpoint of prevention of hypertension. Individuals showing ACI levels of 194,863 or higher are suspected to be excessive alcohol drinkers (≥22 g/day) and have a high risk of hypertension. The cutoff value of ACI was prepared for discriminating habitual alcohol drinkers (alcohol intake of 22 g/day or more) who have a risk of hypertension. The cutoff values of ACI for heavy drinkers or hazardous drinking were higher than that (194,863) for the prevention of hypertension (ACI: 219,467 [heavy drinkers] and 310,679 [very heavy drinkers]).

Regarding multicollinearity among MCV, GGT, and HDL-C, Spearman’s rank correlation coefficients were 0.153 (*p* < 0.01) between MCV and GGT, 0.138 (*p* < 0.01) between MCV and HDL-C, and −0.182 (*p* < 0.01) between GGT and HDL-C. Although these coefficients were statistically significant, there were no remarkable differences in the degrees of the correlations among the three biomarkers. Moreover, the correlations were much weaker than the correlations between each variable of combined biomarkers (except for the product of MCV and HDL-C) and alcohol consumption as shown in Table 3. Therefore, the multicollinearity is unlikely to influence the comparison of the variables of the combined biomarkers. Smoking is positively associated with GGT levels [16,17] and is inversely associated with HDL-C levels [18,19]. The trends of the relationships between alcohol consumption and GGT and HDL-C levels, as well as the relationships between alcohol consumption and ACI, were confirmed to not be influenced by smoking in the multivariable analyses with adjustment for a habit of smoking (Table 2, Figure 3).

The sensitivity and specificity of ACI for excessive alcohol intake were approximately 75%, meaning that both the false positive and negative rates were approximately 25% and this test caused one-fourth of the wrong estimates. The positive predictive value was 69.2%. Thus, a considerable number of cases of excessive alcohol intake may have been misidentified using ACI, although ACI showed a strong association with alcohol consumption. When higher cutoffs (44 or 66 g/day) were used for the classification of excessive drinkers, the AUCs for the relationship between ACI and excessive drinking were not larger and the positive predictive values were much lower than those estimated using the lower cut off (22 g/day) of excessive alcohol consumption. Therefore, it can be concluded that the false-positive rate and false-negative rate were 24.9% and 25.5%, respectively, and were too high for using ACI to discriminate individuals with high alcohol consumption correctly. Therefore, ACI is unlikely to be useful as a diagnostic tool, but may have a potential role in longitudinal monitoring, screening, or epidemiological surveillance. Further studies are warranted to investigate the possibility of using the combined index for the above purposes.

### 4.2. Comparison with Previous Studies

In a previous study by Shaper et al. [7], indices of combining markers were calculated using a formula prepared by using the discriminant analysis technique, and the following formula was used for the combination of GGT and HDL-C: log(GGT) + 1.98 × log(HDL-C). In the present study, when this formula was used, the results of the association between the combined markers and alcohol consumption were 0.437 (correlation coefficient with frequency of drinking), 0.555 (correlation coefficient with amount of drinking), and 0.801 (AUC in the ROC analysis), which were comparable to the results obtained when a simple product of the markers was used (Table 3).

Carbohydrate-deficient transferrin (CDT), phosphatidylethanol (PEth), and genetic markers have recently been suggested to be new biomarkers for heavy alcohol consumption [20,21,22,23]. In a previous study investigating relationships between recent alcohol biomarkers and daily moderate alcohol consumption in healthy volunteers, AUCs were 0.92 for PEth and 0.82 for CDT [24]. Thus, the AUCs for combined biomarkers such as the product of GGT and HDL-C and that of MCV, GGT, and HDL-C (Table 3) were comparable to the AUC for CDT and were lower than the AUC for PEth. Thus, further studies are also needed to clarify whether a combination of ACI and other markers, including CDT and PEth, is useful for evaluating alcohol consumption.

### 4.3. Limitations

The limitations of this study are as follows. Since the information on alcohol intake was collected by using self-reported questionnaires, there are possibilities of underreporting. This possible information bias indicates that true alcohol consumption cannot be estimated using biomarkers. Since the ACI cutoff value was tested in the same population using logistic regression analysis, there was a lack of external and internal validation, which needs to be performed in further studies. The participants in this study were Japanese men aged 31–70 years. In general, women are known to be more vulnerable to the effects of alcohol than men, which is explained by gender difference in the pharmacokinetics of alcohol due to lower body water content and lower activity of alcohol dehydrogenase in the stomach compared to men [25]. Consequently, daily alcohol consumption for women is recommended to be a half of the amount for men [13]. Therefore, the cutoff of ACI obtained in this study is only applicable for men and further studies are needed to know the cutoff for women. In young university students, no significant correlations were reported between conventional laboratory tests, including MCV, GGT, and HDL-C, and alcohol consumption [26]. Thus, it is possible that the relationship between the markers and alcohol consumption differs according to age. Moreover, in Eastern Asians, polymorphisms of genes encoding alcohol-metabolizing enzymes, alcohol dehydrogenase, and aldehyde dehydrogenase, strongly influence individual alcohol consumption [27,28]. However, information on polymorphism was not included in the database used and was not available in this study. Accordingly, further studies are needed to clarify whether or not ACI is applicable to women, younger individuals or non-East Asian populations. Since this is a cross-sectional study, further prospective studies are also needed to clarify the significance of the biomarkers for alcohol consumption in the prevention of hypertension.

### 4.4. Future Directions

Using conventional and modern biomarkers for the estimation of alcohol consumption, there is a possibility of integration with machine learning models for predictive accuracy in the future. Thus, there is a potential role for ACI in longitudinal monitoring, screening, or epidemiological surveillance through integration with questionnaires and recent biomarkers, which should be clarified in the future studies.

## 5. Conclusions

ACI—an index of the product of MCV, GGT, and HDL-C—showed a strong association with alcohol consumption. However, its sensitivity and specificity and positive predictive value were insufficient for accurate diagnosis of excessive alcohol consumption. Therefore, conventional blood biomarkers are not useful for estimating alcohol consumption to prevent hypertension, even when they are used as a single index after combining. Further studies are warranted to test a possibility of application by integrating ACI with recent biomarkers including CDT and PEth for the estimation of alcohol consumption.

## Figures and Tables

**Table 1:** Characteristics of the participants.

Variable	Number, Proportion, Mean, or Median
Subject number	8172
Age (years)	50 (44, 56)
Frequency of drinking (%)	occasional, 32.2; regular, 38.1
Smoking (%)	light, 32.3; heavy, 6.6
Exercise (%)	14.7
Height (cm)	167.8 ± 8.1
Body weight (kg)	66.7 ± 12.9
BMI (kg/m^2^)	23.62 ± 3.79
Systolic blood pressure (mmHg)	129.6 ± 18.8
Diastolic blood pressure (mmHg)	81.0 ± 12.7
Hypertension (%)	40.2
Erythrocyte count (× 10^4^/μL)	483.3 ± 42.7
Hematocrit (%)	44.0 ± 3.7
MCV (fL)	91.1 ± 5.1
GGT (U/L)	32 (20, 55)
HDL-C (mg/dL)	62.8 ± 16.3

Number, proportion, mean with standard deviation, and median with interquartile range in parenthesis of each variable are shown.

**Table 2:** Comparison of each variable in drinker groups categorized by frequency and amount of alcohol consumption.

Drinker Categories	MCV (fL)	Log (GGT [U/L])	HDL-C (mg/dL)
Nondrinkers	89.51 (89.33–89.70)	1.414 (1.402–1.425)	59.37 (58.81–59.94)
Occasional drinkers	90.73 ** (90.55–90.91)	1.506 ** (1.495–1.516)	61.53 ** (60.99–62.08)
Regular drinkers	92.75 **,## (92.59–92.92)	1.687 **,## (1.677–1.697)	66.58 **,## (66.08–67.09)
Nondrinkers	89.60 (89.41–89.80)	1.408 (1.397–1.419)	59.51 (58.93–60.09)
Light drinkers	91.59 ** (91.25–91.93)	1.545 ** (1.525–1.565)	63.43 ** (62.39–64.46)
Moderate drinkers	93.03 **,## (92.80–93.26)	1.692 **,## (1.679–1.706)	67.53 **,## (66.83–68.24)
Heavy drinkers	93.79 **,## (93.42–94.15)	1.803 **,## (1.782–1.824)	68.87 **,## (67.76–69.97)
Very heavy drinkers	94.34 **,## (93.46–95.22)	1.846 **,## (1.795–1.897)	68.69 **,## (66.01–71.36)

Means with 95% confidence intervals in parentheses are shown. Adjustment was performed for age, body weight, and habits of smoking and regular exercise. Symbols denote significant differences from nondrinkers (**, *p* < 0.01) and occasional or light drinkers (##, *p* < 0.01).

**Table 3:** Relationships between alcohol-related variables and alcohol consumption.

Alcohol-Related Variables	Correlation with Frequency of Alcohol Drinking (n = 8172)	Correlation with Amount of Alcohol Consumption (n = 5540)	AUC in ROC Analysis (n = 5540)
MCV	0.314 (0.294–0.334) **	0.388 (0.365–0.411) **	0.708 (0.695–0.722) **
GGT	0.407 (0.388–0.425) **	0.525 (0.505–0.545) **	0.785 (0.773–0.797) **
HDL-C	0.117 (0.095–0.139) **	0.136 (0.109–0.162) **	0.575 (0.560–0.590) **
MCV × GGT	0.423 (0.405–0.441) **	0.544 (0.524–0.563) **	0.795 (0.783–0.807) **
MCV × HDL-C	0.173 (0.152–0.195) **	0.205 (0.179–0.231) **	0.613 (0.598–0.627) **
GGT × HDL-C	0.451 (0.433–0.469) **	0.574 (0.556–0.592) **	0.811 (0.800–0.822) **
MCV × GGT × HDL-C	0.466 (0.448–0.483) **	0.590 (0.572–0.607) **	0.819 (0.809–0.830) **

Shown are Spearman’s rank correlation coefficients with 95% confidence intervals between alcohol-related variables (MCV, GGT, HDL-C, and their combinations) and frequency and amount of alcohol consumption, and the AUCs with 95% confidence intervals for the relationships between the alcohol-related variables and excessive amount of alcohol consumption (22 g/day or more) in the ROC analysis. Significant correlation coefficients and AUCs are indicated with asterisks (**, *p* < 0.01). When analyzing the amount of alcohol consumption and excessive alcohol consumption, occasional alcohol drinkers were excluded from the analysis.

## Data Availability

The data that support the findings of this study are available from the corresponding author (I.W.) for researchers upon reasonable request.

## Abbreviations

The following abbreviations are used in this manuscript:

ACI	Alcohol consumption index
ANCOVA	Analysis of covariance
AUC	Area under the ROC curve
BMI	Body mass index
CDT	Carbohydrate-deficient transferrin
GGT	Gamma glutamyl transferase
HDL-C	High density lipoprotein cholesterol
MCV	Mean corpuscular volume
PEth	Phosphatidylethanol
ROC	Receiver-operating characteristics
